# Role of Liraglutide Use in Patients With Heart Failure

**DOI:** 10.7759/cureus.50065

**Published:** 2023-12-06

**Authors:** Sanjana Allam, Sahil Sartaj, Hiba Moquim, Muhammad Ammar Husnain, Daniel Bustos, Mohit Lakkimsetti, Avneet K Randhawa, Ishita Gupta

**Affiliations:** 1 Internal Medicine, Gandhi Medical College, Secunderabad, IND; 2 Internal Medicine, Melmaruvathur Adiparasakthi Institute of Medical Sciences and Research, Melmaruvathur, IND; 3 Internal Medicine, Shadan Institute of Medical Sciences, Hyderabad, IND; 4 Internal Medicine, Combined Military Hospital Lahore Medical College and Institute of Dentistry, Lahore, PAK; 5 Internal Medicine, Pontifical Catholic University of Ecuador, Quito, ECU; 6 Internal Medicine, Mamata Medical College, Khammam, IND; 7 Internal Medicine, Maharishi Markandeshwar Institute of Medical Sciences and Research, Ambala, IND; 8 Internal Medicine, Dr. Rajendra Prasad Government Medical College, Tanda, Kangra, IND

**Keywords:** glucagon-like peptide-1 receptor agonist (glp-1 ra), review of clinical trials, management of heart failure, glp-1 analogs, literature review, pub-med indexed research, heart failure with preserved ejection fraction, heart failure with reduced ejection fraction, liraglutide

## Abstract

Heart failure is a clinical condition in which the heart is unable to maintain adequate cardiac output. Liraglutide is a glucagon-like peptide 1 (GLP-1) analogue that is used for the treatment of type 2 diabetes mellitus, but recent evidence suggests that it might have a beneficial role in treating heart failure. We conducted a review of existing literature and found five relevant studies. Data from these studies were extracted and then extrapolated into results following analysis. Four of the five studies found an increase in heart rate in heart failure patients. All five studies reported an increased rate of hospitalization. The five studies also showed an increased risk of adverse effects such as arrhythmia, ventricular tachycardia, atrial fibrillation, and worsening of heart failure. Given the scarcity of evidence in the available literature on liraglutide in heart failure, more research on this population is required.

## Introduction and background

Heart failure

Heart failure is defined as a clinical syndrome caused by either structural and/or functional damage, leading to the heart's inability to maintain adequate cardiac output [1,2]. From a public health perspective, it represents a significant clinical challenge as it is a very common cause of hospitalizations and is associated with hospital mortality rates ranging from 4% to 11%, particularly among individuals over the age of 65. This, in turn, translates into a greater financial burden on healthcare systems [3-5]. Moreover, its prevalence is similar in different regions of the world, with figures such as 1.8% in the United States, 1.9% in Canada, and a range of 1% to 2% in Europe in the general population [6].

The classification of heart failure varies and depends on the clinical presentation and the percentage of left ventricular ejection fraction (LVEF). Echocardiography is used to determine this aspect, leading to the categorization into three different groups: heart failure with preserved ejection fraction (HFpEF) (LVEF >50%), heart failure with midrange ejection fraction (HFmrEF) (LVEF 40-49%), and heart failure with reduced ejection fraction (HFrEF) (LVEF <40%), allowing the differentiation to know the treatment and future prognosis [4].

Clinical symptoms can vary depending on the patient's stage and may include congestive symptoms such as dyspnea, orthopnea, lower limb swelling, and jugular venous pulsation; these symptoms help differentiate between a "dry" or "wet" stage. Additionally, it is essential to consider peripheral perfusion symptoms like cold extremities, oliguria, and a narrow pulse pressure to distinguish between a "cold" and "warm" patient, which aids in clinical classification [2]. The purpose of this document is to share relevant information for daily practice, which may also be valuable for future research.

Glucagon-like peptide-1 (GLP-1) agonists

Glucagon-like peptide-1 (GLP-1) is an incretin peptide released by intestinal L cells [7]. This hormone is known for its capacity to augment insulin synthesis and secretion from pancreatic beta cells when there’s an increase in glucose levels [8]. It also carries out additional functions, such as suppressing glucagon secretion, slowing gastric emptying, stimulating beta cell proliferation, and inhibiting appetite, which ultimately leads to reduced food intake [8]. GLP-1 agonists have gained recognition for their effectiveness in enhancing glycemic control and improving cardiovascular outcomes among diabetes patients with an increased cardiovascular risk [9]. In addition, they also have a promising role in managing obesity [9]. The following seven drugs are currently being used in practice: exenatide, lixisenatide, liraglutide, exenatide, dulaglutide, and semaglutide, of which liraglutide and semaglutide have shown promising cardiovascular benefits [10,11]. Common adverse effects associated with this class of medications encompass nausea, vomiting, diarrhea, headaches, nasopharyngitis, injection site reactions, and the potential for acute kidney injury [12,13].

Liraglutide

Liraglutide is a glucagon-like peptide-1 (GLP-1) analogue that is administered once a day in the treatment of type 2 diabetes mellitus. Liraglutide results in the reduction of glucose levels in the blood (both fasting and postprandial), as well as the slowing of gastric emptying and enhancing glucose-dependent insulin secretion [14].

Liraglutide is used in the treatment of obesity by maintaining lifestyle-induced weight loss changes through inhibition of appetite. A university in Denmark tested liraglutide once a day in combination with moderate-intensity exercise and a trial low-calorie diet of 800 kcal per day. Results from the trial showed a weight loss of 5% minimum from their initially recorded baseline weight, along with a mean decrease of -9.5 kg (95% CI, −13.1 to −5.9; P<0.001) in the group who were treated with an exercise program along with once-a-day liraglutide [15]. This study conducted in 2015 on the effects of 3.0 mg liraglutide found a reduction in hemoglobin A1c (HbA1c), glucose levels (fasting and postprandial), and an increase in insulin and C-peptide levels when compared to a placebo. These effects were also recorded as a reduction in prediabetes, blood pressure, and C-reactive protein at week 56 between the liraglutide and placebo groups. The study summarizes these therapeutic effects of liraglutide as being associated with a higher quality of life through patient questionnaires [15]. A double-blinded trial compared the efficacy of liraglutide against orlistat in a multi-institution trial conducted in Europe. The trial participants underwent an exercise program and were given one of four liraglutide doses once a day, three doses of orlistat daily, or a placebo once a day. The doses of liraglutide used were 1.2 mg, 1.8 mg, 2.4 mg, or 3.0 mg. The study found that 76% of the patients enrolled in the trial lost more than 5% weight when on 3.0 mg of liraglutide compared to the patients on placebo. It was also noted that a high prevalence of enrolled patients (>85%) saw reduced blood pressure and blood glucose levels at doses of 1.8 mg to 3.0 mg [16]. The Step 8 clinical trial conducted in 2022 sought to evaluate and compare the mean weight loss of liraglutide to that of another novel GLP-1 agonist. Among the 338 enrolled participants, patients on liraglutide experienced a weight loss of 6.4% [17].

Liraglutide has also been tested in children as an adjuvant to metformin monotherapy in a double-blinded randomized clinical trial conducted over 26 weeks. In 134 patients who were being treated with metformin (with or without insulin), two groups were separated into those treated with 1.8 mg liraglutide and those with a placebo. The main outcomes examined in this trial were fasting plasma glucose levels and mean glycated hemoglobin. These two outcomes represent a standard measure for estimating glycemic control. Over 52 weeks, the addition of liraglutide was observed to have improved glycemic control [18]. Other potential uses of liraglutide that are being examined include renal conditions such as diabetic kidney disease, in which the primary outcomes to improve would be eGFR and albuminuria [19], as well as cardiovascular conditions that could lead to strokes, myocardial infarctions, and potentially cardiac death [20,21].

Liraglutide is a relatively newer drug, so the side effects are yet to be fully understood. Some side effects that have been discovered through a clinical trial include mild to moderate gastrointestinal distress (specifically nausea and vomiting in the first four to eight weeks of treatment) [15,17], gallbladder-related complications (such as cholecystitis and cholelithiasis that were treated by elective cholecystectomy in 75% of patients experiencing these side effects), pancreatitis, reduced mean resting pulse rate (associated with arrhythmias), and spontaneous hypoglycemia [15]. Gastrointestinal adverse events have been reported in many trials, for example, in over 80% of the patients in the Step 8 trial [17]. Coincidentally, patients who experienced gallbladder-related complications also experienced a greater mean decrease in weight [15]. The Step 8 clinical trial started their trial participants at a dose of 0.6 mg/day and increased it to 3.0 mg/day over the initial four weeks of the trial [17]. More trials and research will elucidate the most ideal dosing mechanism for patients to avoid most of the known side effects of liraglutide.

Glucagon-like peptide-1 agonists in heart failure 

Although many people across the globe use GLP-1 agonists for diabetes management since they mimic the naturally produced glucagon-like peptide-1, in recent years there has been increasing evidence suggesting they might have a beneficial role in heart failure too [22]. This is mainly due to their anti-inflammatory effects, improved endothelial function, and effects on heart rate. As a result of this, it prevents cardiac remodeling and decreases myocardial hypertrophy as well as myocardial oxygen demand [23]. Short-term studies have revealed that, in addition to the aforementioned effects, the GLP-1 analogues have also shown positive effects on weight loss and blood pressure regulation, which in turn leads to better outcomes in heart failure [24]. To sum it up, all of these effects together are the underlying reason for the reduced hospitalization rates of heart failure patients also taking GLP-1 analogs. Moreover, the results of the large liraglutide effect and action in diabetes: evaluation of cardiovascular outcome results (LEADER) trial and other studies strengthened the very idea that the use of liraglutide (a GLP-1 agonist) was associated with a significant decrease in cardiovascular mortality compared to placebo [22].

The gap in the existing literature

GLP-1 agonists are frequently used in treating diabetes, partly due to their cardiovascular effects, which also serve as an added benefit. However, certain aspects of this pharmacological group remain untapped, such as the effective cost of GLP-1 agonists compared to the already present standard of care. In addition, there is still limited knowledge on how these cardiovascular benefits vary among the GLP-1 agonists themselves since much of the research, though not all of it, is centered around liraglutide, semaglutide, and incretin-based drugs only. Another point to consider is that if any cardiovascular benefit exists, then the optimal dosing and administration of GLP-1 agonists in different populations with a spectrum of different co-morbidities in addition to type 2 diabetes mellitus is still an unexplored path. We conducted an in-depth review and found a few clinical trials on liraglutide use in heart failure.

## Review

Methodology

Search Methods and Strategy

To conduct a comprehensive literature review on the role of GLP-1 agonists, most importantly liraglutide, in heart failure management, we diligently skimmed through electronic databases like PubMed and clinicaltrials.gov. The search strategy period was conducted between October and November 2023.

The search terms were mindfully constructed to capture all relevant studies. They included a combination of keywords such as “liraglutide,” “GLP-1 agonists,” “heart failure,” “cardiac dysfunction,” “efficacy,” “safety,” “mortality,” “hospitalization,” and “cardiovascular outcomes.” The Boolean operators “AND” and “OR” were included to combine search terms and enhance the search strategy’s effectiveness. We limited our searches to clinical trials, human studies, and controlled or randomized trials. Only publications in the English language have been included. We evaluated all the results that met the inclusion criteria. Two authors retrieved and read each study. Screening and extraction of information from each article were done independently by two authors. A senior author resolved any disagreements through discussion and mutual agreement.

Data Screening and Eligibility

We included articles that met the following criteria for inclusion: (1) double-blinded randomized controlled trials published in PubMed-indexed journals; (2) studies done with human participants and not animal models; (3) studies including adult participants only; (4) studies that included patients diagnosed with heart failure with left ventricular ejection fraction (LVEF)≤ 45%; (5) studies involving the use of liraglutide as a treatment for heart failure; (6) studies that are published in English only.

We excluded articles based on the following criteria: (1) in vitro studies; (2) observational studies, case series, case reports, systematic reviews, and meta-analysis; (3) studies including pediatric participants; (4) studies that did not use liraglutide for treating heart failure; (4) articles published in foreign languages.

By doing so, we included five articles [25-29] in our study. Table 1 lists all the studies included in our review.

**Table 1:** Summary of all included studies

Title of the study	First author	DOI
Effects of liraglutide on cardiovascular outcomes in patients with diabetes with or without heart failure [25]	Steven P.Marso	https://doi.org/10.1016/j.jacc.2019.12.063
Risk of adverse events with liraglutide in heart failure with reduced ejection fraction: a post hoc analysis of the FIGHT trial [26]	Joao Sérgio Neves	https://doi.org/10.1111/dom.14862
Effects of liraglutide on clinical stability among patients with advanced heart failure and reduced ejection fraction [27]	Kenneth B. Margulies,	https://doi.org/10.1001/jama.2016.10260
Heart rate increases in liraglutide treated chronic heart failure patients: association with clinical parameters and adverse events [28]	Rasmus Stilling Tougaard	https://doi.org/10.1080/14017431.2020.1751873
Effect of liraglutide, a glucagon-like peptide-1 analogue, on left ventricular function in stable chronic heart failure patients with and without diabetes (LIVE)—a multicentre, double-blind, randomized, placebo-controlled trial [29]	Anders Jorsal	https://doi.org/10.1002/ejhf.657

Data Collection and Analysis

We extracted data whenever available in different categories, such as paper identifiers (title, DOI, country, date of publication), demographics (age, sex, number of participants in drug and placebo groups), parameters looked at during the study, results, and conclusion of the studies.

We classified the study based on heart failure with either a preserved or reduced ejection fraction. The data was tabulated using Microsoft Excel (Microsoft Corporation, Redmond, Washington, United States). We referenced the paper using EndNote (Clarivate, London, United Kingdom) and followed the guidelines.

Since the data was obtained from already available databases and patients were not directly involved in this study, ethical approval was not needed.

Results 

Demographic Analysis

From the information retrieved, a total of five studies that met the eligibility criteria were included [25-29].

The number of participants included in our review was 10,422 in total from all the studies.

One of the five studies did not mention the number of male and female participants; hence, the sex ratio presented here excludes the study [28]. Excluding 241 participants from the study [28], it left us with a total of 10,181 participants, out of which 3680 (36%) were females and 6501 (64%) were males.

All participants were above 18 years of age, with an age range of 30-85 years and a median age range of 61-66 years from all the studies [25-29].

One out of five studies was a multinational study [26], two out of five were conducted in the United States [26-27], and the remaining two were based in Denmark [28-29].

Two out of the five studies were conducted over 24 weeks [26,29], while the remaining three were conducted over a fairly longer duration of two to five years [25,27,28].

We summarise the demographic data in Table 2 below.

**Table 2:** Demographic analysis

Total number of participants	Age range	Number of participants taking liraglutide	Number of participants on placebo	Sex	
				Males	Females
10,422	The mean age range of the participants was 61-66 years; all participants were above 18 years of age	5220	5202	6501(64%)	3680(36%)

Drug, Dosage, and Type of Heart Failure 

According to the reviewed studies, liraglutide was primarily compared to a placebo [25-29]. 

Most studies used a maximum dose of 1.8 mg per day [25,27,28]. In some cases, an initial dose of 0.6 mg was started and subsequently increased to 1.2 mg and then to 1.8 mg after two weeks [26, 29].

In the majority of studies, the study population consisted of patients with heart failure and reduced ejection fraction (HFrEF) [26-28]. In one study, the assessment of ejection fraction was not taken into account for heart failure patients; rather, the New York Heart Association (NYHA) classification was utilized for evaluation [25].

Effects of Liraglutide on Heart Failure

An increase in LVEF was recorded in only one of the five articles included in this review [29]. However, a statistically significant difference was not noted in the absolute increase in the LVEF among the patients in the liraglutide group and those in the placebo group (mean difference: −0.9%; (95% CI: −2.1, 0.3); P = 0.15). Furthermore, a statistically insignificant change in systolic blood pressure (delta estimate: −2 (−6, 3); P = 0.45) was also recorded in one study [29].

Heart rate change was recorded in four of the articles included in our study [25,27-29]. The article on the LEADER trial [25] reported an increase in heart rate in patients in the liraglutide group against the placebo group, but no statistically significant difference was present among treatment groups or heart failure history. Similarly, two of the articles on the LIVE trial [28,29] reported an average 6±9 bpm. increase among the patients under the liraglutide group and a 1±8 bpm. decrease in those under the placebo group (mean difference: 7 bpm.; (95% CI: 5,9); P <0.0001). These articles also established a dose-related change in heart rate in the liraglutide group. On the other hand, one study comprising the functional impact of GLP-1 for heart failure treatment (FIGHT) trial [27] reported no statistically significant difference in the change of heart rate (mean change in heart rate: −1.6 (95% CI: −4.8, 1.6); P = 0.33).

Similarly, two of the studies [27,29] reported a change in the N-terminal pro-B-type natriuretic peptide level from the baseline levels. However, no statistically significant difference was observed among the liraglutide and placebo groups, with a mean difference of -155 pg/mL and -140 pg/mL, respectively.

Adverse Effects

Although hospitalization has been recorded in all five articles [25-29] included in our study, three articles [25,28,29] demonstrated an association between hospitalization and liraglutide usage. The rest of the articles [26,27] observed a statistically insignificant difference in hospitalization between the liraglutide and placebo groups. Where one article [26] reported on the total events of heart failure hospitalization (incidence rate ratio: 1.47 (95% CI: 0.98, 2.20); P = 0.061), the other article [27] mentions heart failure rehospitalization (hazard ratio: 1.30 (95% CI: 0.89, 1.88); P = 0.17).

Death was surveyed in four studies included in our review [25-27,29]. The study on the LEADER trial [25] did not report any statistically significant difference in all-cause mortality among the two treatment groups (hazard ratio: 0.85 (95% CI: 0.74, 0.97); P = 0.63). Two studies on all-cause death in the FIGHT trial [26,27] reported a statistically insignificant difference among the two treatment groups (incidence rate ratio: 1.10 (95% CI: 0.57, 2.14); P = 0.78). Similarly, only one death was reported in the liraglutide group compared to no deaths in the placebo group in the study on the LIVE trial [29].

Other adverse outcomes such as arrhythmia, ventricular tachycardia, atrial fibrillation, worsening of heart failure, and acute coronary syndrome were reported in all five articles included in our review [25-29]. Three articles [26,27,29] established a statistically significant risk of adverse outcomes in patients under the liraglutide group against the placebo group with (hazard ratio: 1.41 (95% CI: 1.01, 1.97); P = 0.043), (treatment effect: 1.34 (95% CI: 1.00, 1.80); P = 0.05), and (liraglutide: 10% (n = 12); placebo: 3% (n = 3); P = 0.04), respectively. Whereas, one article [25] reported a statistically insignificant difference in major adverse cardiovascular events among the liraglutide and placebo groups (hazard ratio: 0.87 (95% CI: 0.78, 0.97)). Similarly, one article that dealt with heart rate changes in the LIVE trial [28] demonstrated a statistically significant decrease (p<0.0001) in the QT interval in the liraglutide group (22 ± 27 ms) when compared with the placebo group (0.8 ± 24 ms). Furthermore, this study reported a statistically insignificant difference (P = 0.13) among the two groups in the ventricular tachycardia episodes, with liraglutide increasing the episodes from 1.0 ± 3.0 to 1.8 ± 4.0 per patient and placebo decreasing the episodes from 1.7 ± 4.0 to 1.0 ± 2.4 per patient per six months.

Other Relevant Findings

The six-minute walking distance was measured in two articles included in our study [27,29]. While one article found a statistically significant increase in the liraglutide group (mean difference: 24 m (95% CI: 2, 47); P = 0.04) [29], the other article reported a statistically insignificant increase in the liraglutide group (treatment effect: 5 (95% CI: -29, 39); P = 0.79) [27]. Another article included in our study reported a statistically insignificant association between the increase in heart rate and the six-minute walk distance [28].

The findings from one article [26] suggest that in the liraglutide group, a statistically significant increase (P = 0.008) in the risk of adverse effects was seen in NYHA classes III and IV. This study also suggests a statistically significant increase (P = 0.051) in the risk in heart failure patients comorbid with diabetes. This article also established that patients with two or more heart failure hospitalizations presented with a statistically significant increase in severe-symptomatic heart failure compared to those with a single or no heart failure hospitalization (P = 0.050). It also noted a statistically significant decrease in the LVEF with subsequent hospitalizations (P = 0.016) and more frequent usage of cardiac devices (P = 0.048).

Table 3 illustrates a summary of our findings. The parameters have been listed in alphabetical order.

**Table 3:** Qualitative analysis CI: confidence interval; p-value: probability value; ms: millisecond

Parameters	Findings
Adverse outcomes	A significant risk of adverse outcomes was established in patients under liraglutide compared to the placebo group (hazard ratio: 1.41 (95% CI: 1.01, 1.97); P = 0.043), (Treatment effect: 1.34 (95% CI: 1.00, 1.80); P=0.05), (liraglutide: 10% (n = 12); placebo: 3% (n = 3); P = 0.04), respectively [26,27,28].
Six-minute walking distance	A significant increase in the six-minute walking distance was reported in the liraglutide group (mean difference: 24 m (95% CI: 2, 47); P = 0.04) [29].
Heart rate changes	An increase in heart rate was recorded in the liraglutide group compared to the placebo group ((mean difference: seven beats per minute; (95% CI: 5.9); P < 0.0001)) [28,29].
QT interval changes	A significant decrease (p < 0.0001) in the QT interval was observed in the liraglutide group (22 ± 27 ms) compared to the placebo group (0.8 ± 24 ms) [28].
Ventricular tachycardia episodes	No significant difference was found between the groups in ventricular tachycardia episodes (P = 0.13) [28].

Discussion 

Summary of Our Results

This review aimed to investigate and compare the effects of liraglutide against placebo in heart failure patients. A total of five articles were included, encompassing 10,422 participants [25-29]. All patients were aged 18 years and older, spanning a range from 30 to 85 years. The duration of the studies ranged from 24 weeks to five years.

Some of the significant findings were as follows: a remarkable increase in heart rate was observed with the use of liraglutide, and a majority of the studies reported increased incidences of hospitalization. All articles documented a range of adverse effects, such as arrhythmias, worsening heart failure, and acute coronary syndrome, with the intake of liraglutide.

No significant changes were observed in terms of LVEF, systolic blood pressure (SBP) readings, N-terminal pro-brain natriuretic peptide (NT-proBNP) levels, or the occurrence of death.

Liraglutide in Heart Failure 

Though various glucose-lowering drugs are available for the treatment of diabetes mellitus, many patients still struggle to attain therapeutic outcomes, and cardiovascular complications continue to be a primary cause of mortality among this patient population. In search of medications that have better cardiovascular risk reduction, liraglutide is being increasingly considered. Individuals undergoing treatment with liraglutide have experienced a notable reduction in the occurrence of cardiovascular-related deaths, nonfatal strokes, nonfatal myocardial infarctions, and overall mortality [30]. The LEADER trial also made a significant observation that liraglutide-treated patients exhibited lower hospitalization rates due to heart failure in comparison to the placebo group [25]. The benefits observed in these studies indicate a compelling potential for liraglutide in treating diabetic patients with high cardiovascular risk.

A study found that liraglutide increased heart rate by 2.71 bpm (1.45 to 3.97) versus placebo [31]. This was in alignment with our findings from the LIVE trial, which reported a 6±9 bpm increase in the liraglutide group versus a 1±8 bpm decrease in the placebo group.

On the other hand, our analysis also revealed contradictory results compared to various articles. While a few articles have suggested a reduction in major adverse cardiovascular events (MACE) by 14%, all-cause mortality by 12%, and hospital admissions by 11% with the use of liraglutide [30,25,32], our review identified reports of various adverse cardiovascular events, higher rates of hospitalization, and no discernible decrease in all-cause mortality.

Other and Newer GLP-1 Agonists in Heart Failure

Diabetes mellitus and heart failure are two highly prevalent comorbid conditions that are commonly interconnected in their pathology and insidious nature. A statement from the Heart Failure Society of America and the American Heart Association details how the prevalence of diabetes mellitus is higher in cohorts of patients with heart failure, regardless of their ejection fractions. This increase in the prevalence of heart failure among patients with diabetes is between 9% and 22%, with an increased prevalence among diabetic patients over 60 years old [33]. The Heart Failure Association of the European Society of Cardiology says that there is a frequent association between type 2 diabetes mellitus and heart failure, with 30-40% of heart failure patients being burdened with type 2 diabetes mellitus. Type 2 diabetes mellitus can lead to many adverse effects on the coronary blood flow and directly on the myocardium [34]. The prevalence of both type 2 diabetes mellitus and heart failure has only continued to worsen, both in the United States and globally [35].

GLP-1 agonists are a novel tool in a physician’s arsenal for combating diabetes mellitus. The GLP-1 receptor agonist classification is categorized similarly to the classification of types of insulin: short-acting or long-acting based on how long they synthetically activate the GLP-1 receptor. These include lixisenatide and exenatide (short-acting), liraglutide, dulaglutide, and albiglutide (long-acting), with exenatide also available in a long-acting form [36].

A meta-analysis conducted in 2021 examined eight trials where GLP-1 agonists were trialed to understand their cardiovascular outcomes. The authors of this meta-analysis found that there was a 14% lower risk of major adverse cardiovascular events (MACE) in patients with concurrent comorbidities of diabetes mellitus and a history of cardiovascular disease. This meta-analysis included a total of 60,080 patients with type 2 diabetes mellitus. When the study results were stratified based on the presence of prior cardiovascular disease, it was found that GLP-1 agonists had a reduction of 6% in the risk of MACE in patients without a history of cardiovascular disease. The three components of MACE studied by this meta-analysis were nonfatal myocardial infarction (MI), nonfatal stroke, and cardiovascular mortality [37]. Type 2 diabetes mellitus can also be considered a risk factor for cardiovascular mortality in itself, and thus this compounding beneficial effect of GLP-1 agonists requires more in-depth understanding. Through the analysis of eight clinical trials, GLP-1 agonists were found to have reduced all-cause mortality by 12% and the risk of heart failure by 10% [37].

Another meta-analysis conducted in 2019 examined 18 randomized clinical trials to compare the efficacy of GLP-1 agonists to a currently adopted standard of treatment, sodium-glucose cotransporter-2 inhibitors (SGLT2i). The MACE examined includes the previously mentioned, along with hospitalization and renal outcomes. Over 77,242 patients were included, and their results found that both GLP-1 agonists and SGLT2i had a similar mean reduction in MACE, with 12% in GLP-1 agonists and 11% in SGLT2i. However, their similarities stopped when examining hospitalization and renal outcomes. SGLT2i was responsible for a 31% reduction in hospitalizations due to heart failure. As for renal outcomes, the primary outcomes studied included estimated glomerular filtration rate (eGFR) and microalbuminuria. Only SGLT2i reduced the risk of worsening eGFR, whereas both drug classes reduced the risk of progressive microalbuminuria (GLP-1: 0.82 p<0.001, SGLT2i: 0.62 p<0.001) [38]. The results of this meta-analysis show the proven benefits of adding a GLP-1 agonist to the regimen, perhaps in addition to established gold treatment standards. When observing patients with distinct atherosclerotic histories, the visible therapeutic effect of GLP-1 agonists rises to a 14% risk reduction. The metric used in this study was the hazard ratio (HR) [38]. A 2023 analysis examined a cohort of older adults with heart failure (HFrEF or HFpEF) from Medicare data to compare the adjusted hazard ratios among those taking SGLT2i vs. GLP-1 agonists vs. DPP4i. There was no significant difference in the risk of MI or stroke among those taking SGLT2i or GLP-1 agonists [39].

GLP-1 agonists carry favourable benefits and reduce risk across many systems. Eligible trial data related to cardiovascular outcomes taken from four trials (LEADER - liraglutide, SUSTAIN 6 - semaglutide, EXSCEL - exenatide, ELIXA - lixisenatide) was analyzed in 2018 to find a 10% reduction in the relative risk of MACE. 10% can be considered significant [40].

Other Ongoing Studies

Ongoing studies investigating incretin-based therapies for cardiovascular health have both similarities and distinctions. Four of these studies focus on the outcomes of GLP-1 receptor agonists (RAs) or dipeptidyl peptidase 4 (DPP-4) inhibitors [41-44]. The semaglutide treatment effect in people with heart failure with preserved ejection fraction (STEP-HFpEF) study stresses the obesity phenotype of heart failure with preserved ejection fraction; it investigates the outcomes of semaglutide in this patient group [41]. Comparably, the exenatide study of cardiovascular event lowering (EXSCEL) study investigates the effect of exenatide on cardiovascular outcomes in patients with type 2 diabetes mellitus and various cardiovascular risk profiles, leading to a better understanding of GLP-1RAs in cardiovascular health [42]. The mechanistic trial explains the effects of incretin-based therapies on cardiovascular, gastrointestinal, and renal systems in subjects with type 2 diabetes mellitus [43]. In comparison, the Semaglutide cardiOvascular oUtcomes triaL (SOUL) study focuses on patients with type 2 diabetes mellitus and established atherosclerotic heart disease and chronic kidney disease, evaluating the cardiovascular risks associated with oral semaglutide [44]. Another study explores the potential effects of liraglutide on left ventricular ejection fraction in chronic heart failure patients, giving insight into the use of GLP-1 analogs in chronic heart failure [45]. 

Limitations

Although studies have been performed on the use of glucagon-like peptide-1 (GLP-1) agonists in heart failure and our paper has summarized all of these into a single-to-go academic reference, like all other educational work, our work also has certain limitations. To begin with, the lack of vast knowledge on this topic prevented us from performing a meta-analysis on the subject. Another reason was that, with already limited data on this topic, even that data had little difference in the essence of what they found. To add to this, liraglutide was the only main representative of the effects of GLP-1 agonists under study. The other GLP-1 agonists were left out of most of the trials and studies we included in our analysis. Thirdly, the studies included in our analysis studied the effects of GLP-1 agonists in heart failure with reduced ejection fraction, taking only those patients into account that had an ejection fraction of less than 45%. All in all, in addition to all of the above, the fact that we did not analyze the studies that compared the effects of these GLP-1 agonists with the already-present standard of care used in the management of heart failure, such as angiotensin-converting enzyme (ACE) inhibitors and beta-blockers, etc., shines a light on the limitations of our study.

## Conclusions

Due to the vast knowledge already present about the standard of care used to treat heart failure and the contraindications that sometimes limit their use, there was a need to analyze newer drugs that could potentially improve the outcomes of heart failure in patients with and without other concomitant conditions, allowing the development of a drug that could be used widely. Taking this into account, our study aimed to analyze different clinical trials done on a glucagon-like peptide-1 agonist, namely liraglutide, to understand its effects on the various outcomes of heart failure and to realize its potential as a pharmacological therapy for heart failure in patients with and without other comorbidities. Various cardiovascular markers were assessed, and it was seen that there was an increase in heart rate in patients taking liraglutide compared to placebo. Given the paucity of data, we feel that further large-scale double-blind randomized controlled trials should be conducted. Moreover, studies analyzing the long-term safety profile and benefits of liraglutide in heart failure using first-hand patient data and comparing it with the current standards of care should also be undertaken.
